# Land use change and Ecological Network in rapid urban growth region in Selangor region, Malaysia

**DOI:** 10.1038/s41598-024-67294-1

**Published:** 2024-07-16

**Authors:** Tian Zi Ma, Bor Tsong Teh, Mei Ye Kho

**Affiliations:** 1 Centre for Sustainable Urban Planning and Real Estate (SUPRE), Faculty of Built Environment, Universiti Malaya, 50603 Kuala Lumpur, Malaysia; 2 The Centre for Building, Construction and Tropical Architecture (BuCTA), Faculty of Built Environment, Universiti Malaya, 50603 Kuala Lumpur, Malaysia

**Keywords:** Ecology, Ecological networks

## Abstract

Rapid urbanization will cause various land use changes and the vast occupation of green spaces, a critical factor in the deterioration of biodiversity in urbanized areas. Some species of wildlife are endangered due to habitat shrunk and fragmentation. However, Malaysia's current biodiversity protection range is still limited. The Ecological Network (EN) refers to a framework of ecological components, which can be obtained by geographical and technical approaches to support more ecological diversity ranges. Furthermore, little research has been found on EN in Malaysia and the impact of land use change on EN. Therefore, the Selangor region is selected as the study area. This paper quantifies land use change and measures the extent of land use change to obtain the EN’s change. The result has shown that forestland has decreased, explored by people for housing and agriculture from 2000 to 2020. The EN has a trend of fragmentation. Overall, this study's results imply that the land use change led to EN's worsened performance from 2000 to 2020 in the study area. This paper hopes that this research could help supply information on conserving biodiversity in future development and urban sustainable planning in Malaysia.

## Introduction

Rapid urbanization has taken place worldwide during the last decades, which resulted in an unprecedented scale and rate of urban expansions and led to fundamental changes in land use and a vast amount of green space occupation around the globe, especially in developing countries^1,2^. The United Nations has predicted that by 2050 about 64% of the developing country and 86% of the developed country will be urbanized (Urban life: Open-air computers. The Economist. 27 October 2012). Continuous urban development means that more green space will be taken up for agriculture or human settlement in the future^3^. As a result, these land use changes, including urbanization, deforestation, and increased agriculture, cause the fragility of habitat patches which providing survival habitat space for wildlife species^3,4^. This phenomenon has several side effects such as habitat loss, fragmentation, worse connectivity for wildlife species, loss of biodiversity, the extinction of some wildlife species, numerous environmental problems, as well as negative impacts on human well-being of citizens^5^.

The Sustainable Development Goal 15 (SDG15) was proposed to restore and promote sustainable use of terrestrial ecosystems, sustainably manage forests, combat desertification, and halt and reverse land degradation and halt biodiversity loss. Although Malaysia has also enacted many policies and regulations promoting SDGs for many years, some species of wildlife are still considered endangered due to habitat shrunk and fragmentation in Malaysia (https://www.mybis.gov.my/pb/590). Therefore, under the background of urbanization, balancing biodiversity protection against regional land use change regulation has become a challenge.

According to the Wikipedia Encyclopedia and Oxford Bibliographies, an Ecological Network (EN) is a representation of the biotic interactions in an ecosystem, in which species (nodes) are connected by pairwise interactions (links). The new paradigm of EN is an inevitable choice to conserve biological diversity, particularly in fragmented landscape^6^. The EN refers to a network connected by scattered habitat patches and crisscrossing ecological corridors, which can be identified as a practical approach to protecting wildlife habitats and improving habitat connectivity in certain regions^7^. The EN idea is derived from national ecological network programs enacted in the 1980s in Central and Eastern Europe^8^. Since the 1980s, the EN program and study in Western Europe, North America, Latin America, Australia, and Asia were launched to integrate protected areas into more extensive, interconnected networks to protect regional biodiversity^8^.

However, few studies exist on EN in Malaysia and the relationship between land use change and EN. Most previous studies mainly focused on ecological connectivity and footprint in Malaysia^4,9,10^, and three aspects of the EN in other regions, i.e., construction, assessment, and optimization. The EN construction scheme immediately shows the ecological performance in a particular area^11,12^. The assessment of EN provides a scientific and reasonable basis for the scientific management of ecological spaces in highly urbanized regions^13,14^. The optimization of EN can provide reliable instructions for spatial conservation and restoration of ENs and enrich the theoretical research of urban biodiversity conservation planning and ecological restoration practice^15,16^. There needs to be more dynamic analysis of EN within the context of understanding the land use change process and transition. This research gap will provide a more rational and visual representation of EN alterations brought on by land use change. It also will supplement the government's construction of ecological spaces to protect biodiversity.

In terms of methodologies, the majority of prior research has relied on land use transition matrix data to illustrate the extent of land use changes^17^. However, few studies have integrated this data with EN changes to uncover the influence of land use on EN changes. Additionally, most existing researches identify the core areas of EN based on species indicators and sociotope/biotope maps^18^, designated natural protected areas^12^ or sites based on case study^19^. It is essential to use a mathematical and scientific approach to identify the core areas of EN. The Morphological Spatial Pattern Analysis (MSPA) is rooted in the principles of mathematical morphology^20^. It utilizes pixels as the fundamental unit for computation to identify core areas that significantly influence the overall connectivity of the EN^21^. Landscape connectivity denotes the ability of species to move and thrive within ecological patches^22^. This paper integrates MSPA and landscape connectivity to eliminate subjectivity in the selection of core areas and enhance the extraction of core areas based on structural attributes, thereby improving their function and connectivity. In the context of EN’s corridor extraction, numerous studies have explored the use of the Minimum Cumulative Resistance (MCR) model^23–25^and circuit theory^26,27^. The MCR model leverages the cumulative lowest cost of consumption to maximize the spread and reflect potential accessibility. However, it may encounter issues such as corridor redundancy and uneven layout^25^. Circuit theory, inspired by the physics of electron flow in a circuit, is utilized to model the migratory movement of biological flows through a heterogeneous landscape^28^. Many studies have employed circuit theory to identify the pinch points and barriers of EN rather than getting ecological corridors^28,29^. On the other hand, the Least-Cost Path (LCP) model identifies the least-cost pathway between habitat patches, possessing comprehensive knowledge of the landscape and providing an optimal pathway for species migration dispersal^30^. This model is considered a rational and easily operated method for designing corridors. Hence this paper combines MSPA, landscape connectivity and LCP model to ensure the scientific validity of EN.

Selangor takes the lead as the most urbanized state in Malaysia. The level of urbanization, which stood at 88.4% in 2005, matched some of the more developed countries^31^. Meanwhile, the Selangor region (Selangor, Kuala Lumpur, and Putrajaya) is the most populated in Malaysia, with a population of about 9.21 million (Department of Statistics Malaysia 2022). There are tensions between land development and ecological integrity in the Selangor region as more land is used (https://www.mybis.gov.my/one/). The biodiversity has faced severe threats such as deforestation, habitat loss, and worse connectivity for wildlife species in this region. In addition, there is a lack of research on EN in the Selangor region, which is essential to highlight the fragility of habitat patches to land use change and to protect biodiversity. Therefore, choosing the Selangor region as the study area, this study aims to investigate the impact of land use change on ecology. This paper quantifies land use change and measures the extent of land use change to obtain the regional EN’s change through GIS. This study will improve awareness of EN to protect ecological diversity and the impact of land use change on EN in Malaysia in the future.

## Study area and data sources

### Study area

**Figure 1:**
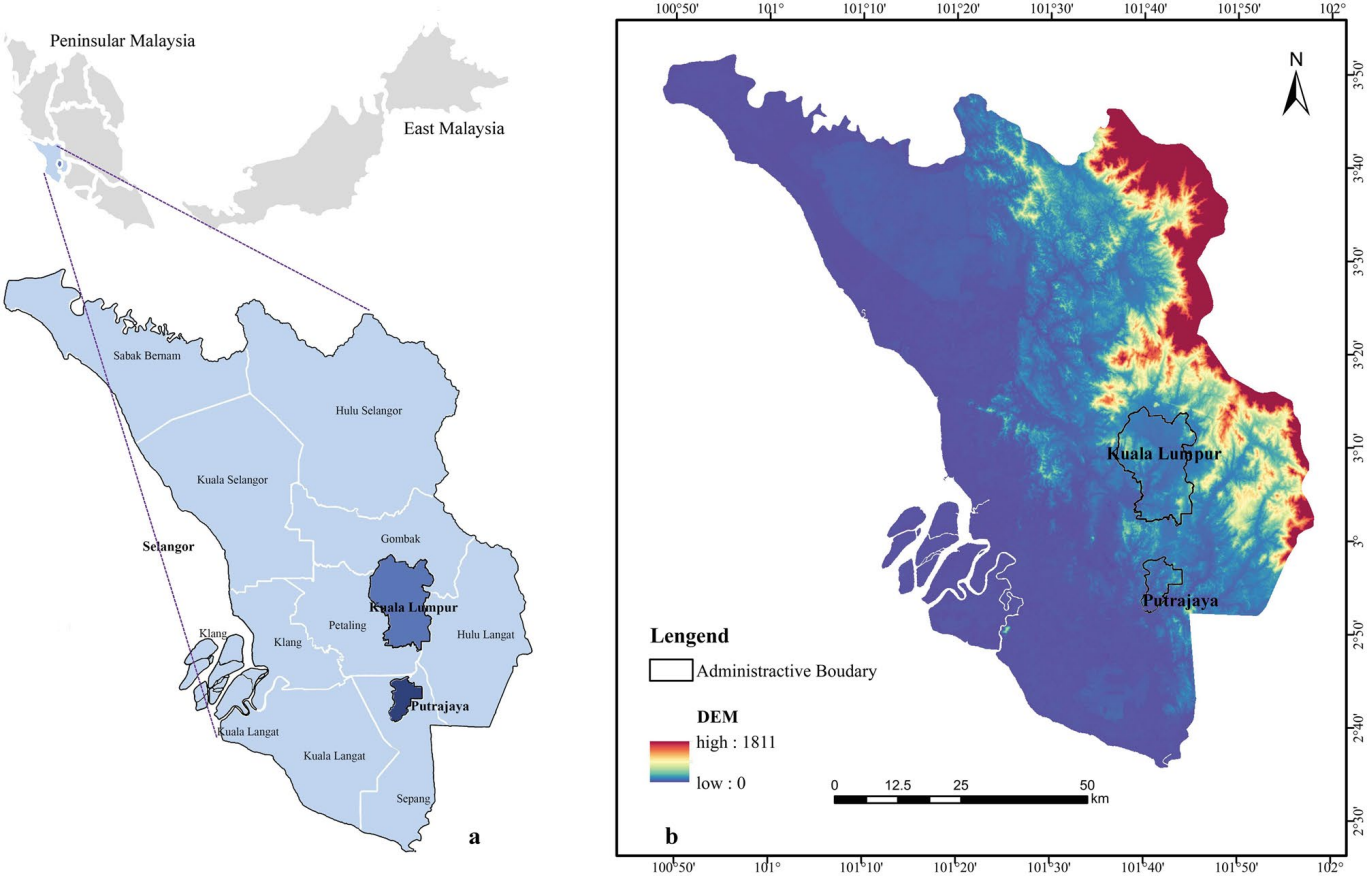
Location of the study area. **a**. Selangor region in Peninsular Malaysia; **b**. administrative map and DEM (created by ArcMap, version 10.8, http://www.esri.com/; Photoshop CC 2019, https://www.adobe.com/).

This study chose the Selangor region comprising Selangor, Kuala Lumpur, and Putrajaya as the study area (Fig. 1). The Selangor region is a finance, cultural, administrative, and other activities hub and has a weighty responsibility for Malaysia's ecological biodiversity. The Selangor region has undergone dramatic economic and population growth. Selangor region's GDP accounted for 38.55% of the national total in 2021 (Department of Statistics Malaysia Official Portal); By 2020, it accounted for 27.91% of Malaysia's population (Department of Statistics Malaysia 2022). In addition, the portion of the built-up area increased dramatically, and the number of living quarters increased to 2686.5 k in 2010^32^. Land use conversion is also becoming more aggressive in this region because the development pressure, whether for construction, agriculture, or logging, threatens green spaces. Controlling land and its natural resources has been a significant opportunity for the Selangor region. Therefore, making people recognize the impact of land use change and the importance of EN is a prerequisite for protecting green spaces and regional biodiversity.

### Source of data

The data used in this study mainly include land use datasets, the slope, the Relief Degree of Land Surface (RDLS) and the Digital Elevation Model (DEM), and the Enhanced Vegetation Index (EVI). Considering the uniformity of the data, obtaining several sets of complete land use data in the study area is necessary, and the data time interval should be the same. However, only the GlobeLand30 (http://www.globallandcover.com/) has complete sets of data (30 m resolution) for the study area^33^. The DEM data (90 m resolution) were obtained from the Shuttle Radar Topography Mission (SRTM) product (https://www.gscloud.cn/), and the slope and the RDLS data were calculated from the DEM data through ArcGIS10.7 software. The EVI data were downloaded from https://www.earthdata.nasa.gov/.

### Data preprocessing and validation

All original data was standardized using the WGS_1984_UTM_Zone_47N projected coordinate system and WGS84 coordinate system to ensure consistency. Subsequently, the DEM data is preprocessed by utilizing the Resample tool in ArcToolbox within the ArcGIS 10.7 software. This will allow for achieving a consistent resolution with other types of data. Additionally, it's important to adjust the Environment setting in ArcGIS software to ensure that all data pixels correspond one-to-one, and to eliminate any position offset.

The land use data provided by GlobeLand30 is a globally accessible 30-m resolution land cover dataset developed by the National Geomatics Center of China^34^. The overall accuracy of GlobeLand30 has been evaluated at 80.63%^33,35^. At the same time, multiple representative sample points were selected from the dataset to validate the accuracy. The accuracy of the land use classification data was verified using the confusion matrix, and it exceeded 82%. Based on this validation, it can be concluded that GlobeLand30 is a dependable product suitable for a wide range of applications.

## Research methods

**Figure 2:**
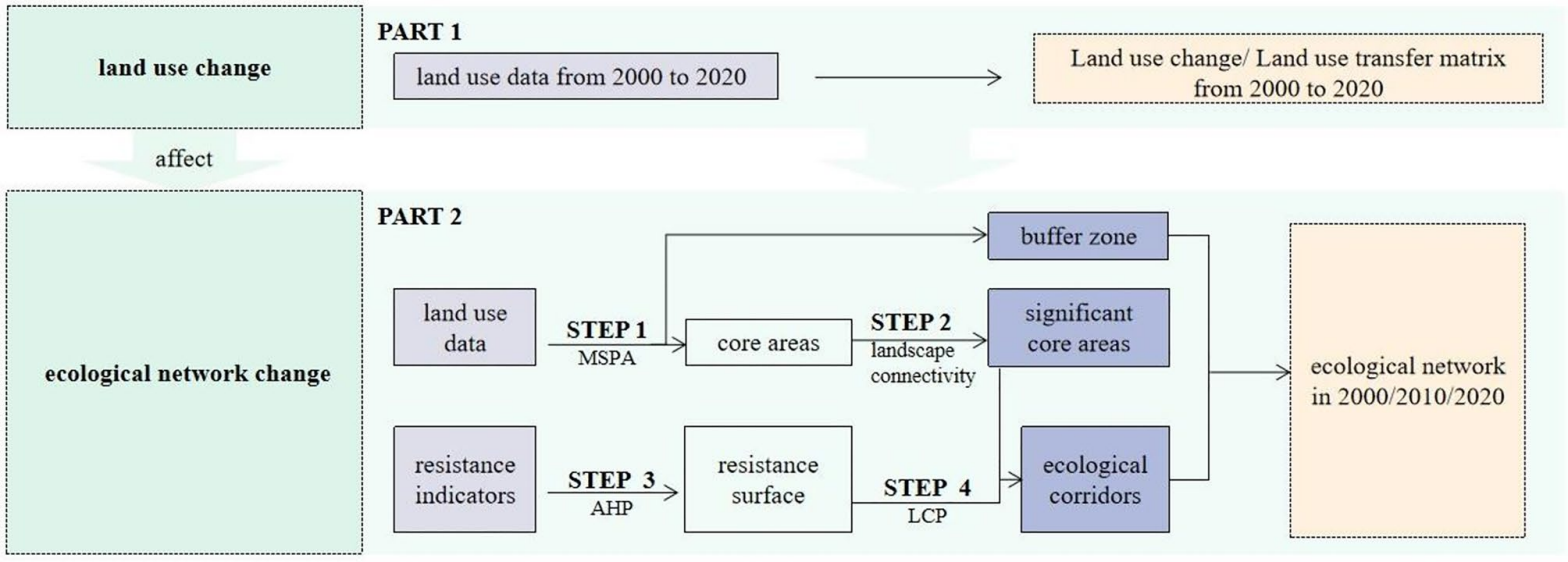
Workflow and methods used to identify land use change and the dynamic of EN.

According to Fig. 2, firstly, this paper quantifies land use change, measures the extent of land use change, and gets the land use transition matrix (PART 1).

Secondly, according to the land use data from 2000 to 2020, this part tries to get specific EN (2000–2020) which consists of core areas, buffer zones, and corridors (PART 2).

### Land use change

The land use change degree of a single land use type, commonly represented as $K$, means the quantity change of a particular land use type within a specific time range in a specific research area. It can be calculated using the following formula^36,37^:

1 $$\begin{array}{c}K=\frac{{U}_{b}-{U}_{a}}{{U}_{a}}\times \frac{1}{T}\times 100\%\end{array}$$

${U}_{a}$ and ${U}_{b}$ are the area of land use types in the early and late stages, respectively, and $T$ is the time interval between the two periods.

The comprehensive land use change degree is often expressed by ${L}_{\text{C}}$^36,37^, which refers to the overall situation of various land use types in a specific research area within a certain period of time. It can be calculated using the following formula:

2 $$\begin{array}{c}{L}_{\text{C}}=\frac{{\sum }_{i=1}^{n}\Delta {L}_{{\text{U}}_{i-j}}}{2{\sum }_{i=1}^{n}\Delta L{\text{U}}_{i}}\times \frac{1}{T}\times 100\%\#\end{array}$$

$\Delta {L}_{{\text{U}}_{i-j}}$ is the absolute value of the area of the $i$ land use type converted to a non-$i$ land use type during the monitoring period; $\Delta L{\text{U}}_{i}$ is the area of $i$ land use type at the monitoring start time; $T$ is the time interval between two periods.

The land use transition matrix can quantitatively indicate the conversion between different land use types in two periods, reflecting the changes in the spatial and temporal patterns of land in a specific region^38^. The calculation of the land use transfer matrix is based on the following formula:

3 $$\begin{array}{c}{K}_{\text{ab}}=\left|\begin{array}{cccc}{k}_{11}& {k}_{12}& \cdots & {k}_{1n}\\ {k}_{21}& {k}_{22}& \cdots & {k}_{2n}\\ \vdots & \vdots & & \vdots \\ {k}_{s1}& {k}_{s2}& \cdots & {k}_{sn}\end{array}\right|\#\end{array}$$

${K}_{\text{ab}}$ is the area from the $\text{a}$ land use type in the initial period to the $\text{b}$ land type in the final period; $n$ is the number of land use types.

### EN identification

As Fig. 2 shows, EN comprises core areas, buffer zone, and corridors. Getting core areas and buffer zone: (STEP 1–2) Identifying the focal species is a critical step to help determine the MSPA and corridor procedure parameters. Then MSPA is combined with landscape connectivity to identify ecological core areas and buffer zone^23,39^.

Getting corridors: (STEP 3) According to the relevant literature, resistance surface indicators are chosen. Then, determine the weight of each resistance surface indicator according to the Analytic Hierarchy Process (AHP) and expert scoring to form the resistance surface ^1^. (STEP 4) Ecological corridors were calculated by LCP model based on the resistance surface ^40^.

#### Core areas and buffer zone based on MSPA and landscape connectivity

- Focal species selection

From the landscape connectivity perspective, monitoring and managing every species is unrealistic. One or more species should be chosen as typical species indicators of habitats and corridors.

Moreover, the sambar deer was already in Endangered status in Peninsular Malaysia at the beginning of the twenty-first century^41^. Improving habitat connectivity and protecting habitats for sambar deer has the most significant probability of promoting population reproduction. Meanwhile, the survival of the sambar deer is also pivotal to realizing Malaysia’s goal of saving the Malayan tiger (The Malaysian Conservation Alliance for Tigers). Thus, this study chooses sambar deer, which is sensitive to different vegetation types and urgently needs to be protected as the focal species.

- The Morphological Spatial Pattern Analysis (MSPA)

MSPA can be used for measuring, identifying, and segmenting the morphological patterns of digital raster maps that describe the connectivity of the image components^21^. In addition, the automated recognition of numerous structures in the landscape, which may precisely determine core areas, islets, and other landscape types, is a unique benefit of MSPA^42,43^. In this paper, MSPA can help identify the importance of core areas crucial for getting ENs at the pixel level.

Specifically, the pixels of a digital raster map within the ecological space are referred to as the foreground, while the remaining pixels are considered the background (non-ecological space)^2,42^. Thus, define the primary livable land use type-forestland, wetland, and water body-as our foreground map suited for Sambar Deers living and migrating. Then set other land use types as the background map.

Through GuidosToolbox software (https://forest.jrc.ec.europa.eu/en/activities/lpa/gtb/), seven visually distinguished MSPA classes are obtained: core, islet, perforation, edge, loop, bridge, and branch^21^. Core and islet are all ecological spaces and they can provide habitats or migration places for wildlife^23^. Edges are the external boundary of the core, as a buffer zone between the ecological space and the non-ecological space, which plays a key role in protecting the core area and maintaining the stability of the area^24^. Perforations are the internal boundary of the core; Loops are connecting corridors inside the same core; bridges are connected to the adjacent cores; branches are connected at one end to the edge, perforation, bridge, or loop^42^. However, in this study, the core and islet are regarded as the study area’s core area of EN because they provide spaces for sambar deers to survive and reproduce; the edge is regarded as the buffer zone.

- Landscape connectivity

The probability of connectivity index ($\text{PC}$) is defined as the probability that two animals randomly placed within the landscape fall into habitat areas that are reachable from each other (interconnected) given a set of $\text{n}$ habitat patches and the connections (${\text{p}}_{\text{ij}}$) among them^44^. It is given by the following expression:

4 $$\begin{array}{c}PC=\frac{{\sum }_{i=1}^{n} {\sum }_{j=1}^{n} {a}_{i}\cdot {a}_{j}\cdot {p}_{ij}^{*}}{{A}_{L}^{2}}\#\end{array}$$

where ${a}_{i}$ and ${a}_{j}$ are the areas of the habitat patches (core areas) $i$ and $j$, and ${A}_{L}$ is the total landscape area (area of the study region, comprising both habitat and non-habitat patches).

The $\text{d}PC$ (the delta of $\text{PC}$), importance of each habitat patch (core area) in terms of its individual contribution to the maintenance of overall landscape connectivity as measured by dPC^4,44^.

5 $$\begin{array}{c}dPC=\frac{PC-P{C}_{\text{remove }}}{pc}\times 100\%\#\end{array}$$

The dPC value refers to the patch importance index in terms of interpatch connectivity, its intrinsic high habitat quality, or a combination of both factors^45^. The higher the dPC value is, the better patch connectivity and habitat quality perform. Generally speaking, evaluating by dPC can help identify and prioritize the core area of EN that most contributes to overall landscape connectivity for sambar deer on top of the MSPA results^46^.

Therefore, STEP 1 can get a bunch of core areas (cores and islets) by MSPA, which would include alarming connectivity degree in the landscape. Using all these core areas to calculate is impractical. Thus, significant core areas were selected to be larger than 3 km^2^ with the dPC value ≥ 0.1 from 2000 to 2020 instead of the whole core areas from MSPA.

- Calculating resistance surface

Resistance surfaces are indices that characterize the willingness or likeliness of wildlife to migrate through an area, which is mainly related to specific important land surface characteristics^2,23,47^. According to the actual situation of the study area, expert opinion and the relevant literature^26,29,48^, land use type, DEM, slope, EVI, and RDLS were eventually chosen as resistance surface indicators with five classes of value. The lower the resistance value, the more suitable the resistance surface raster is for wildlife migration^23,24^. This study considers forestland and water body as ideal habitats for sambar deer due to their minimal disturbance from human activities; they have been assigned lower resistance values. While wetlands and grasslands are essential for survival, they also can impede migration. On the contrary, built-up areas strongly hinder species migration assigned with high resistance values. Topography is also a critical factor influencing migration, with lower elevations and relatively flat terrain being more conducive to species dispersal. Furthermore, areas with high forest cover provide favorable living conditions for sambar deer with lower resistance values^49^. The AHP model was developed by Saaty^50^, is a multi-objective decision making approach that employs a pairwise comparison procedure to arrive at a scale of preferences among a set of alternatives^51^. The AHP, along with expert scoring, was used to determine the weight of each factor in the resistance surface (Table 1). Finally, comprehensive resistance surfaces were formed through the ArcGIS 10.7 Raster Calculator Tool as a preparation for getting ecological corridors.

**Table 1:** Resistance weights and values.

Resistance indicator	Weight	Classification	Resistance value
Land use	0.38	Forestland/water body	1
		Wetland	3
		Grassland	5
		Cropland	7
		Build-up area/bareland	9
Digital Elevation Model (DEM)/m	0.14	< 120	1
		120–340	3
		340–630	5
		630–980	7
		> 980	9
Slope/(°)	0.18	< 3	1
		3–8	3
		8–15	5
		15–23	7
		> 23	9
Enhanced Vegetation Index (EVI)	0.14	> 0.6	1
		0.5–0.6	3
		0.4–0.5	5
		0.2–0.4	7
		< 0.2	9
Relief Degree of Land Surface (RDLS) /(°)	0.16	< 16	1
		16–40	3
		40–70	5
		70–108	7
		> 108	9

#### Ecological corridors

An ecological corridor is the shortest functional zone of passage between several large patches of habitat, facilitating the spread and migration of animals^6^. The LCP model is a straightforward resistance-based model commonly used to identify areas in a landscape where movement is likely to be favored^30,52^. It evaluates potential animal movement routes across the landscape based on the cumulative ‘cost’ of movement^53^. And Manon Balbi et al. validated the ecological relevance of LCP analysis to identify habitat linkage design, and could be easily implemented by urban landscape planners^30,54^. The Linkage Mapper tool in ArcGIS software can be used to calculate LCP. Subsequently, input maps of core areas and comprehensive resistance surfaces to determine the ecological corridors through Linkage Mapper tool.

## Results

### Land use change

The area of Selangor, Kuala Lumpur, and Putrajaya is 8104 km^2^, 243 km^2^, and 49 km^2^, respectively. Therefore, their sum area is 8396 km^2^. Overall, forestland, built-up area, and cropland were listed in order of their land use area, which consistently ranked in the top three from 2000 to 2020. The forestland accounts for about 59% of the total area. The second land use type was the built-up area, taking up about 20%, and the proportion of cropland was about 15% (Table 2).

**Table 2:** The proportion of different land use type from 2000 to 2020.

Land use type	2000			2010			2020		
	Area/km^2^	Percentage (%)	Rank	Area/km^2^	Percentage(%)	Rank	Area/km^2^	Percentage(%)	Rank
Bare land	0	0		0	0		11.55	0.14	
Cropland	1131.75	13.48	3	1158.16	13.79	3	1485.85	17.70	3
Forestland	5193.00	61.85	1	5101.86	60.77	1	4510.06	53.72	1
Grassland	294.00	3.50		251.00	2.99		186.78	2.22	
Built-up	1478.32	17.61	2	1575.48	18.76	2	1921.21	22.88	2
Water body	99.40	1.18		92.32	1.10		72.73	0.87	
Wetland	199.53	2.38		217.18	2.59		207.83	2.48	

**Figure 3:**
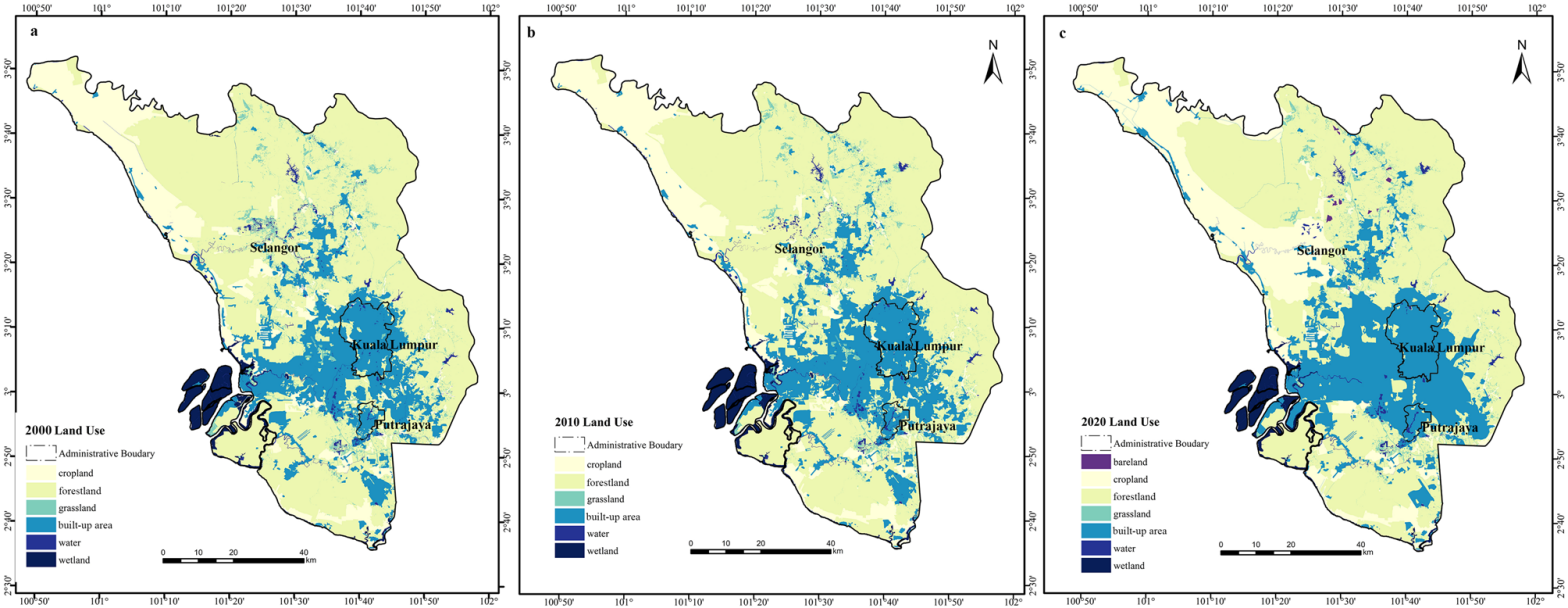
Distribution maps of land use. **a**. 2000; **b**. 2010; **c**. 2020 (created by ArcMap, version 10.8, http://www.esri.com/).

As referred to the Fig. 3 land use distribution map from 2000 to 2020, the distribution density of the built-up area in Kuala Lumpur, Putrajaya, Petaling, Kalang, and Hulu Langat was very high. Meanwhile, the forestland was mainly distributed in Hulu Selangor, Gombak, Hulu Langat, and Kuala Langat. By 2020, a new land use type, bare land, emerged.

In terms of land use change, it can be found that the amount of built-up area had grown the most during the previous 20 years, whereas the amount of forest land had greatest declined throughout the same time, which shows the trend of urbanization. For example, the land use change degree of single land use type was positive, including cropland, built-up area, and wetland, indicating that their area had increased in the last 20 years (Table 3). These three land use type proportions of the total area increased by 4.22%, 5.27%, and 0.10% individually. Among the negative land use change degree (Table 3), forestland, grassland, and water body could be seen, showing that their area had declined from 2000 to 2020; their proportion of total area decreased by 8.13%, 1.28%, and 0.31%.

**Table 3:** Land use change from 2000 to 2020.

Land use change		First decade (2000–2010)	Second decade (2010–2020)	2000–2020	2000–2020 Negative land use change
Land use change degree of single (each) land use type (%)	Cropland	0.23	2.83	3.13	
	Forestland	− 0.18	− 1.16	− 1.32	Declined
	Grassland	− 1.46	− 2.56	− 3.65	Declined
	Built-up	0.66	2.19	3.00	
	Water body	− 0.71	− 2.12	− 2.68	Declined
	Wetland	0.88	− 0.43	0.42	
Comprehensive land use change degree(%)		0.17	0.82	0.97	
The area of the land use change/km^2^(absolute value)		282.43	1369.94	1633.66	

Furthermore, the land use change degree of single land use type in 2010–2020 is much larger than that in 2000–2010. Likewise, it also can be seen from Table 3 that the comprehensive land use change degree and the area of land use change in 2010–2020 had increased significantly compared with that in the previous decade. This feature means that from 2010 to 2020, large-scale land development occurred, and land use changed a lot in the Selangor region.

**Figure 4:**
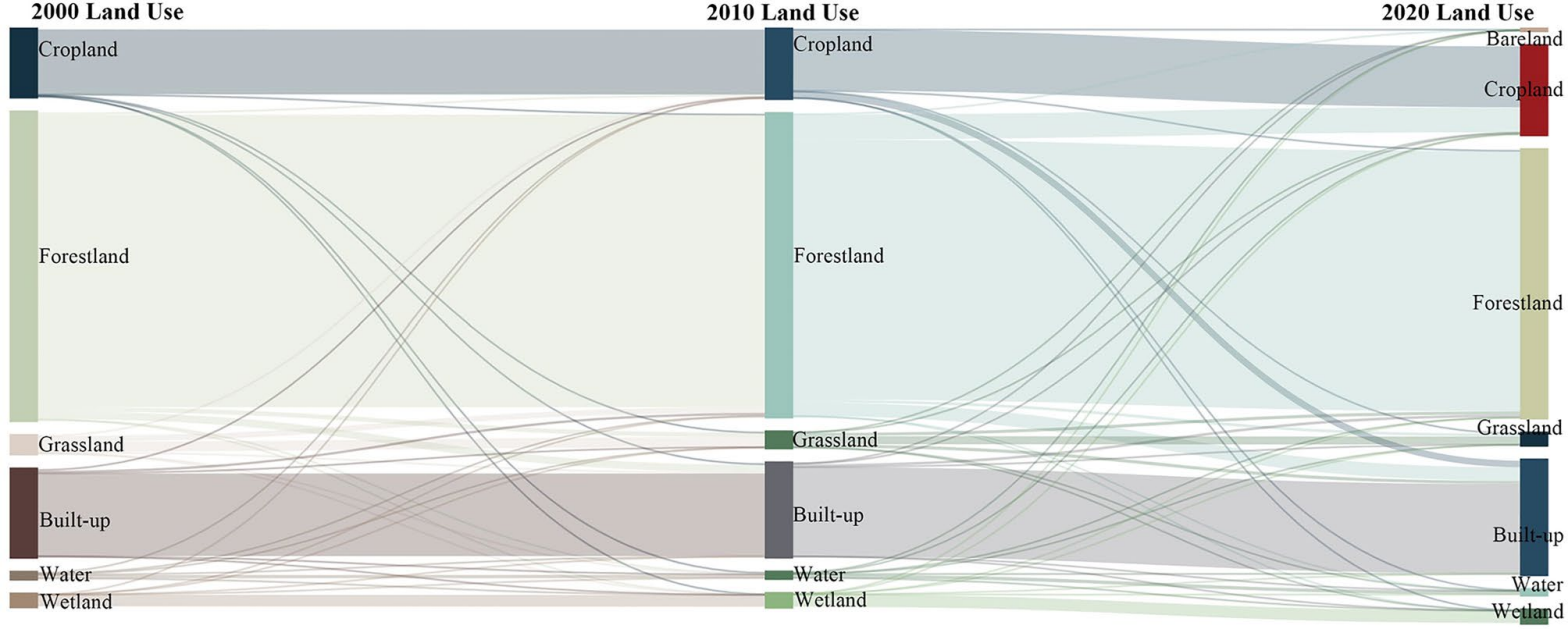
Sankey diagram of land use transition matrix from 2000 to 2020.

In terms of land use transition, the Fig. 4 Sankey diagram visually shows the conversion situation of each land use type. For example, from 2000 to 2010, the total land use change area was 282.43 km^2^. The forestland losses were the largest, mainly converted into the built-up area, and the built-up gains were the largest. However, the wetland losses and gains area were both the smallest. From 2010 to 2020, the total number of land use change was 1369.94 km^2^, and the forestland losses were still the largest, mainly flowing to cropland; the area of cropland and built-up gains were the largest, which mainly conversion from the forest (Fig. 4).

**Figure 5:**
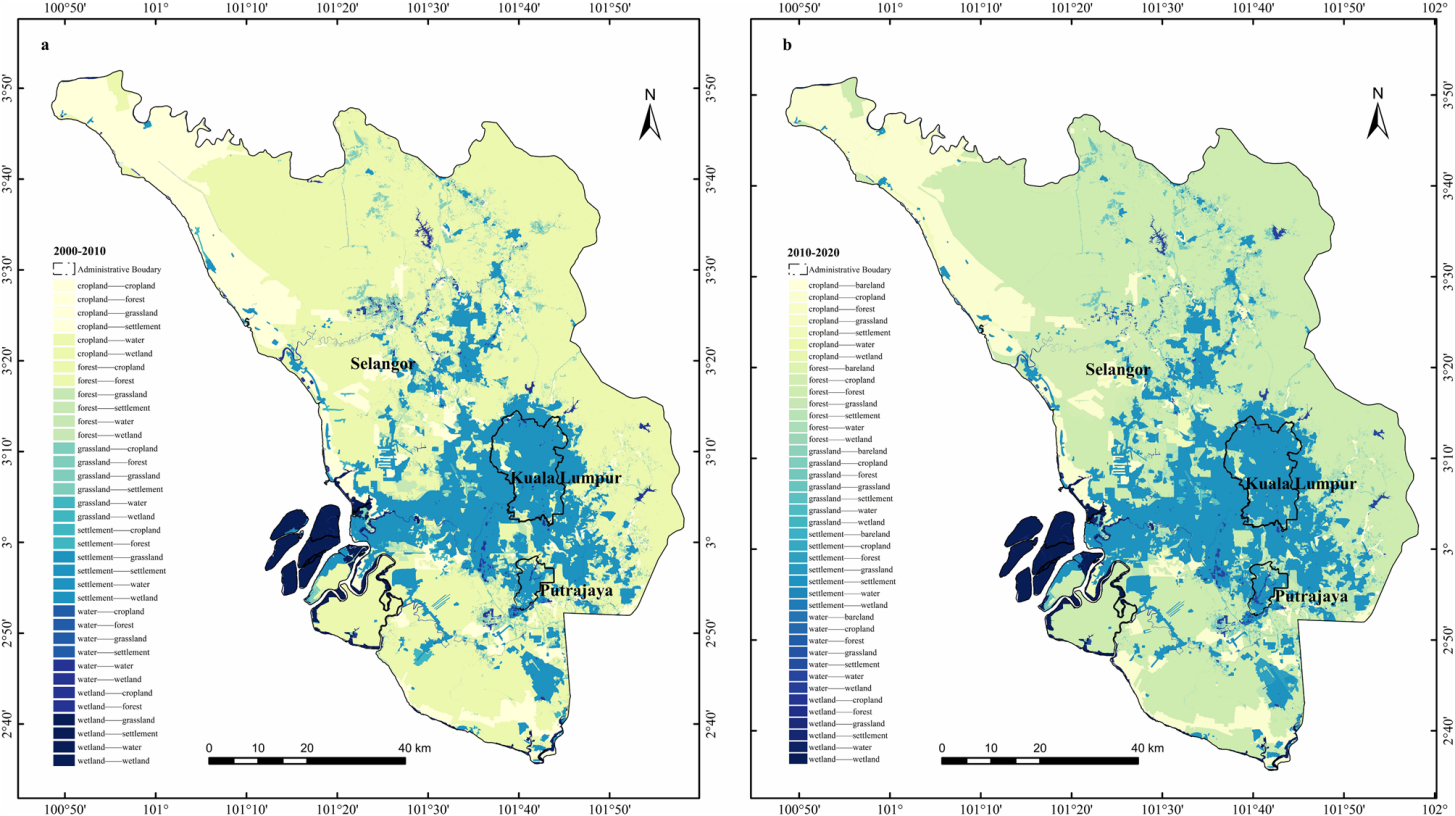
Land use transition matrix distribution map. **a**. 2000–2010; **b**. 2010–2020 (created by ArcMap, version 10.8, http://www.esri.com/).

In summary, the most considerable land use losses occurred in forestland, which mainly converted to cropland and built-up in the past 20 years. According to Fig. 5, deforestation mainly occurred in Sabak Bernam, Kuala Selangor, Kuala Langat, and Sepang for the sake of housing and agriculture. As well as that, the most significant land use gains were built-up. In places where forests had been cut down, a significant human settlement has been created in Kuala Lumpur, Putrajaya, Klang, Petaling, Gombak, Hulu Langat, and Sepang.

### EN change

#### The response of ecological core areas and buffer zone to land use change

The results of calculating ecological and non-ecological space are shown in Table 4. The results indicate that ecological spaces in the study area gradually decreased from 2000 to 2020 due to the increase of cropland and built-up area. In 2000, the ecological space area of the study area was 5491.93 km^2^. However, in 2020 the ecological space area of the study area was 4790.61 km^2^. It shows that with the city's rapid development, the built-up area is constantly expanding, and many green spaces in the study area are occupied.

**Table 4:** Change of ecological spaces from 2000 to 2020.

Ecological space (foreground map)	2000 area/km^2^	2010 area/km^2^	2020 area/km^2^	Non-ecological space (background map)	2000 area/km^2^	2010 area/km^2^	2020 area/km^2^
Forestland	5193.00	5101.86	4510.06	Bareland	0	0	11.55
Water body	99.40	92.32	72.73	Cropland	1131.75	1158.16	1485.85
Wetland	199.53	217.18	207.83	Grassland	294.00	251.00	186.78
				Built-up	1478.32	1575.48	1921.21
	5491.93	5411.36	4790.61		2904.06	2984.64	3605.39

According to Table 5, the EN’s core area and buffer zone were carried out from 2000, 2010, and 2020. The buffer zone area was 301.51 km^2^ in 2000, accounting for 3.59% of the total land use area. In 2020, the area was 271.70 km^2^, accounting for 3.24% of the total land use area. The reduction in buffer zone area indicates that the ability to reduce the degree of interference from human activities is decreasing.

**Table 5:** Core area and buffer zone from MSPA between 2000 to 2020.

		2000 area/km^2^	Percentage of the total area (%)	2010 area/km^2^	Percentage of the total area (%)	2020 area/km^2^	Percentage of the total area (%)
Core area	Core	4437.93	52.86	4434.21	52.81	3961.21	47.18
	Islet	30.59	0.36	23.48	0.28	14.69	0.17
		4468.52	53.22	4457.69	53.09	3975.9	47.35
Buffer zone	Edge	301.51	3.59	295.15	3.52	271.7	3.24

When it comes to the core area, in 2000, it was 4468.52 km^2^, which mostly stayed the same from 2000 to 2010. Nevertheless, a significant reduction from 2010 to 2020 was reduced to 3975.9 km^2^, accounting for 47.35% of the total land use area.

**Figure 6:**
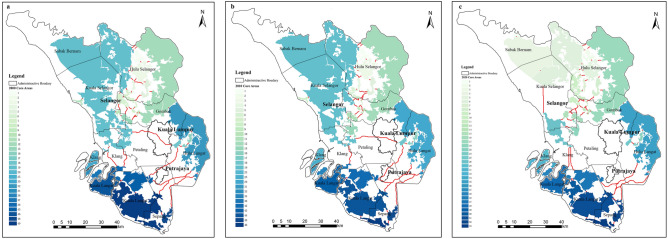
The significant core area and corridor distribution map of the study area. a. 2000; b. 2010; c. 2020 (created by ArcMap, version 10.8, http://www.esri.com/).

As referred to Fig. 6, the significant core areas, those large than 3 km2 with the dPC value ≥ 0.1 are shown. Figure 6 expresses that core areas have been fragmented in the past 20 years. As the continuous urbanization and the area of the core areas decreased from 2000 to 2020, this led to the degree of patch fragmentation in the study area deepened, especially around Kuala Lumpur and Putrajaya.

#### The response of ecological corridors to land use change

Figure 6 presents the ecological corridors from 2000 to 2020. In 2000, 50 ecological corridors with a cumulative length of 270,941 km were identified. 50 ecological corridors were discovered in 2010, totaling 257,234 km. 57 ecological corridors, totaling 217,855 km in length as of 2020, were identified. The result indicates that the number of ecological corridors increased from 2000 to 2020, but the length of corridors decreased. According to Fig. 6, ecological corridors experienced a fragmented tendency from 2000 to 2020. There were corridors passed through Kuala Lumpur and Putrajaya, which were more densely distributed in the middle of the study area. However, by 2020, all corridors in these two regions had disappeared.

## Discussion

This study result indicates conflicts between land development and EN in the Selangor region.

**Figure 7:**
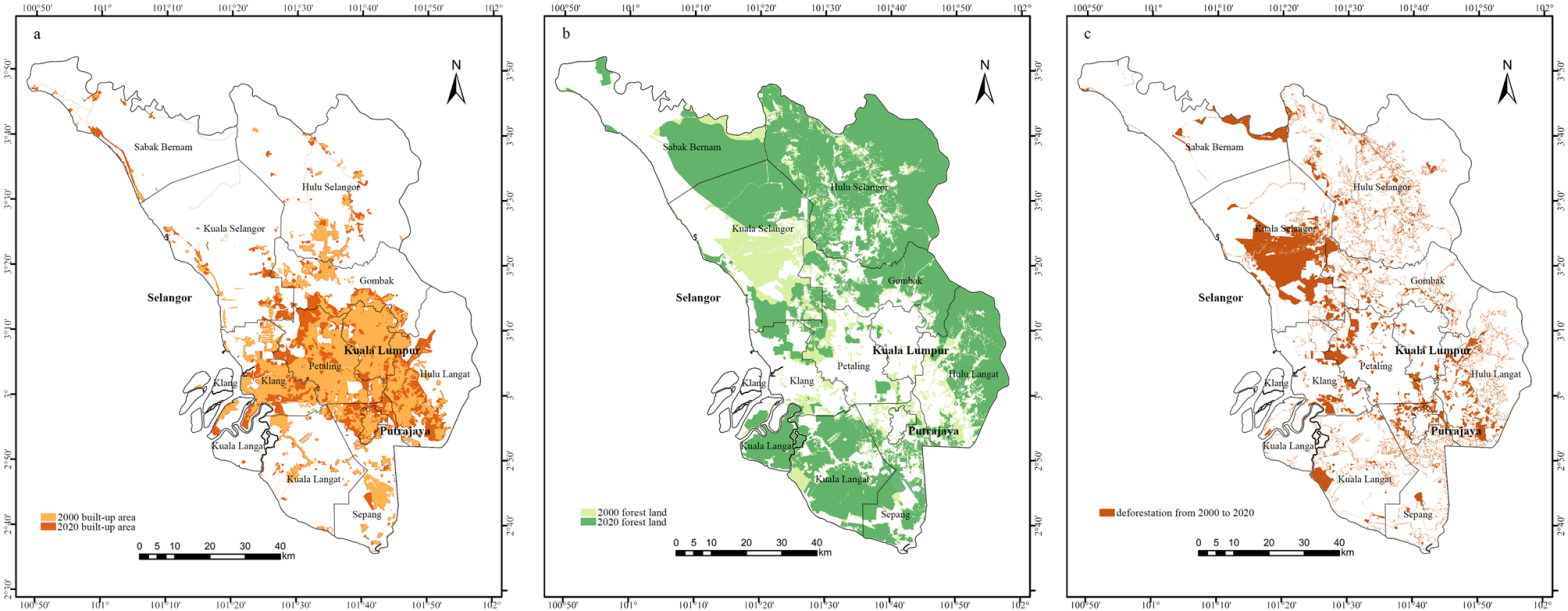
The built-up area (**a**), forestland (**b**) change from 2000 to 2020, and deforestation from 2000 to 2020 (**c**) in Selangor region (created by ArcMap, version 10.8, http://www.esri.com/).

The result of land use change was consistent with the findings of^17^, who found that natural vegetation is turned into build-up and agricultural land cover. This study shows that forestland has decreased considerably (by 8.13%), mainly converted into cropland and human settlement from 2000 to 2020. Although many planning documents have formulated Selangor’s strategic policy to guide and coordinate sustainable urban development (https://www.selangor.gov.my), the data shows that people still develop the economic at the expense of green space^55^. For example, forestland have been cut down mainly in Sabak Bernam, Kuala Selangor, Hulu Langat and Kuala Langat for housing and cropland from 2000 to 2020 (Fig. 7b). In a similar vein, Fig. 7c illustrates that these regions are subject to significant human interference, primarily resulting in deforestation within these areas. Moreover, a significant human settlement has been created in Kuala Lumpur, Putrajaya, Klang, Petaling, Gombak, Hulu Langat, and Sepang (Fig. 7a).

The most likely driving factors for urban expansion are physical and social aspects, such as land use change^3^, population^56^, and economic growth^55^. In addition, the urban expansion and deforestation levels changed significantly compared to other regions in 2000–2020. For example, the built-up area increased 5.27%, and forestland decreased 8.13% in the Selangor region, compared to 1.7% and 2.1% in Johor River Basin^57^. Therefore, township development is necessary to be implemented to ensure no 'urban sprawl' in the rapid growth region. At the same time, visually monitoring land use development in the environment can help control large-scale urbanization.

The result of EN change expresses that EN’s performance gradually worsens due to urban expansion and deforestation, especially around Kuala Lumpur and Putrajaya. This worsening feature means the function of providing survival habitats and good connectivity to immigrate for wildlife in the Selangor region is decreasing^58^. In terms of the significant core areas, they are mainly distributed in the northeast and southwest of the study area, with apparent polarization (Fig. 6). The reasons for this distribution characteristics possibly are: (1) The northeast and southwest of the study area are still largely covered with forests, including protected forests (e.g., Raja Musa Forest, Bukit Tarek Forest, Bukit Gading Forest); (2) Kuala Lumpur and Putrajaya are located in the middle of the study area, a highly urbanized city with few ecological spaces to inform the core areas. Additionally, the prominent patches (significant core areas) became small, and the number of patches increased from 2000 to 2020 due to deforestation; these fragmented patches also led to the number of ecological corridors that providing passages between patches for wildlife immigration increased (Fig. 6). For instance, a significant portion of forestland in Kuala Selangor is occupied, with limited habitat patches available to support biological migration and activities. As a result, a long corridor has emerged in this area. However, the whole length of corridors were declined.

## Future direction and research limitation

**Figure 8:**
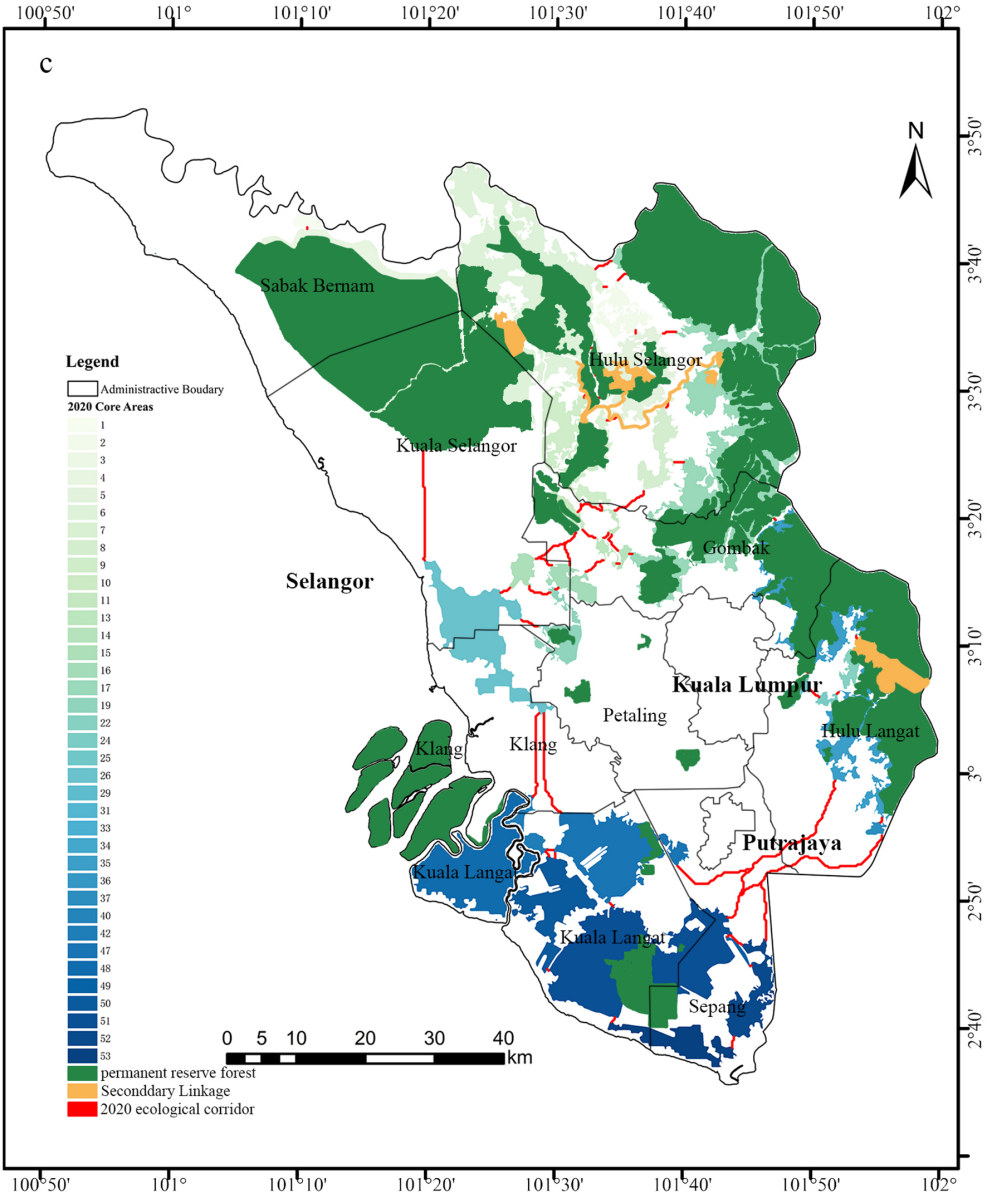
The comparison between EN and CFS in Selangor region (created by ArcMap, version 10.8, http://www.esri.com/; Photoshop CC 2019, https://www.adobe.com/).

Besides that, most previous research concentrated on ecological connectivity^4,59^, forest biodiversity^55^, ecosystem services^60^, and ecological conservation^61^ in Malaysia, and there currently needs to be more studies on EN. In terms of wildlife biodiversity protection policy, as Fig. 8 shows, at present, there are only a few Secondary Linkages (Raja Musa Forest Reserve—Bukit Tarek Forest Reserve—Bukit Gading Forest Reserve) that would be established in the next stage of CFS (https://www.ic-centralforestspine.com.my/publications/) and reserve forests to protect (https://www.mybis.gov.my/one/). However, EN can provide a wide range of structural layouts to protect wildlife habitats and improve habitat connectivity. For example, suppose the government tries to protect the core areas and corridors in Gombak (Fig. 8). In that case, it will bridge a potential linkage for wildlife immigration between the fragmented reserve forests in this region. Alternatively, suppose the government preserves the core areas and corridors in Sepang. In that case, it will allow wildlife to immigrate from Hulu Langat to Kuala Langat rather than without ecological spaces. Therefore, this study can be work as the reference and supplement for government to construct corridors and protect larger range of forests.

When it comes to limitations of this study, they are mainly reflected in the following three points: (1) In terms of the data sources, the majority source of this paper comes from the GlobeLand30 between 2000 and 2020. This is owing to data restrictions and the length of the paper. There may be some errors in the data set. (2) Only one mammal animal was selected as the focal species for the study area; the distance threshold and the connectivity probability of landscape connectivity are set based on this focal species’ migratory capacity. The results may not adequately represent the connectivity needs of some non-focal species, hence future research will require more investigation to verify it. (3) The construction of the resistance surface was necessary for calculating ecological corridors. However, there was no unified standard for selecting resistance indicators and the value of resistance indicators.

## Conclusion

This study was a preliminary attempt to investigate the impact of land use change on EN to improve the awareness of biodiversity protection in the Selangor region. The land use change, land use transition matrix, and EN construction were used to examine the extent of urban expansion and deforestation. This proposed approach helps people realize the impact of land expansion on habitat patches and the importance of balancing economic development and biodiversity protection. The overall results show that a tremendous amount of forestland was explored by people for housing and agriculture. The ecological environment in the study area is declining as EN’s performance gradually worsens and fragments. This study tries to provide a reference for conserving biodiversity in CFSMP in future development and urban sustainable planning in Malaysia. It is recommended to limit the urban sprawl as the city develops its economy to improve the balance between land development and biodiversity protection. In addition, future works may consider developing an extensive range to conserve wildlife biodiversity, like protecting ecological core areas rather than only protected areas and keeping ecological corridors’ connectivity.

## Data Availability

The datasets used and analyzed during the current study are available from the corresponding author on reasonable request.
